# On-Line Evaluation and Monitoring of Security Features of an RO-Based PUF/TRNG for IoT Devices

**DOI:** 10.3390/s23084070

**Published:** 2023-04-18

**Authors:** Luis F. Rojas-Muñoz, Santiago Sánchez-Solano, Macarena C. Martínez-Rodríguez, Piedad Brox

**Affiliations:** Instituto de Microelectrónica de Sevilla, IMSE-CNM, CSIC/Universidad de Sevilla, 41092 Sevilla, Spain; rojas@imse-cnm.csic.es (L.F.R.-M.); macarena@imse-cnm.csic.es (M.C.M.-R.); brox@imse-cnm.csic.es (P.B.)

**Keywords:** physical unclonable functions, true-random number generator, hardware security, key generation, reconfigurable devices, embedded systems

## Abstract

The proliferation of devices for the Internet of Things (IoT) and their implication in many activities of our lives have led to a considerable increase in concern about the security of these devices, posing a double challenge for designers and developers of products. On the one hand, the design of new security primitives, suitable for resource-limited devices, can facilitate the inclusion of mechanisms and protocols to ensure the integrity and privacy of the data exchanged over the Internet. On the other hand, the development of techniques and tools to evaluate the quality of the proposed solutions as a step prior to their deployment, as well as to monitor their behavior once in operation against possible changes in operating conditions arising naturally or as a consequence of a stress situation forced by an attacker. To address these challenges, this paper first describes the design of a security primitive that plays an important role as a component of a hardware-based root of trust, as it can act as a source of entropy for True Random Number Generation (TRNG) or as a Physical Unclonable Function (PUF) to facilitate the generation of identifiers linked to the device on which it is implemented. The work also illustrates different software components that allow carrying out a self-assessment strategy to characterize and validate the performance of this primitive in its dual functionality, as well as to monitor possible changes in security levels that may occur during operation as a result of device aging and variations in power supply or operating temperature. The designed PUF/TRNG is provided as a configurable IP module, which takes advantage of the internal architecture of the Xilinx Series-7 and Zynq-7000 programmable devices and incorporates an AXI4-based standard interface to facilitate its interaction with soft- and hard-core processing systems. Several test systems that contain different instances of the IP have been implemented and subjected to an exhaustive set of on-line tests to obtain the metrics that determine its quality in terms of uniqueness, reliability, and entropy characteristics. The results obtained prove that the proposed module is a suitable candidate for various security applications. As an example, an implementation that uses less than 5% of the resources of a low-cost programmable device is capable of obfuscating and recovering 512-bit cryptographic keys with virtually zero error rate.

## 1. Introduction

The Internet of Things (IoT) has brought about a revolutionary change in the way we live today. With an increasing number of devices connected to the network and exchanging information to access a huge number of applications of a very different nature, guaranteeing the integrity and privacy of these data has become an important requirement. Unfortunately, many IoT devices have been proven to be vulnerable to cyberattacks that have had significant consequences, such as data breaches [1,2,3,4] or interruptions in critical services [5,6,7]. The lack of proper authentication and authorization protocols to protect IoT devices can facilitate unwanted access to sensitive information, phishing by unauthorized parties, and even allow attackers to take control of devices. Another major concern related to this issue is the shortage of efficient procedures for tracking potential security issues, making it difficult to detect and respond to them on time.

To address the aforementioned security shortcomings, hardware-based solutions are becoming an increasingly popular trend. These security solutions are designed to provide strong security features that are physically safe and difficult to manipulate. In contrast to pure software-based cybersecurity, hardware security primitives are immune to software vulnerabilities such as malware or viruses, making them good choices for protecting and saving sensitive or critical data. In addition, because many of these blocks are specially designed to accelerate cryptographic operations, hardware-based solutions offer better timing performance and more effective results than their software-based counterparts. They are also easy to integrate with other hardware pieces, which facilitates the development of secure IoT devices as high-functionality embedded systems [8].

A basic concept behind some hardware-based security solutions for IoT devices is the Root of Trust (RoT). A hardware RoT includes a set of components that help establish trust and prevent safety violations, specially designed to provide a secure basis for system operation and to guarantee authenticity and integrity in data processing and storage [9].

Among the different modules that can build a RoT for IoT devices, Physical Unclonable Functions (PUFs) and True Random Number Generators (TRNGs) are of special interest. On the one hand, PUFs exploit unique and unpredictable features of hardware devices to generate cryptographic material, thus acting as an identifier generator and offering a secure key storage solution. On the other hand, TRNGs provide random numbers for cryptographic operations and other randomization-based processes. The combination of PUF and TRNG functionalities into a single design can provide a higher level of security with minimal hardware requirements when added to the RoT of embedded processors and provide a tamper-proof solution. Furthermore, while ensuring energy efficiency, its dual functionality can be used to build secure applications on devices with limited resources.

Regarding cost-effective and efficient solutions, and considering that IoT devices are often limited in resources, reconfigurable devices such as Field-Programmable Gate Arrays (FPGAs) and programmable Systems-on-Chips (SoCs) have established themselves as technological allies to perform specific functions in a compact and efficient manner, which makes them suitable for portable and battery-powered devices for usages in which power consumption is a critical factor. Two additional features that make FPGAs and SoCs suitable for IoT devices are that they offer scalability and flexibility to be reprogrammed on site, significantly shortening development time and maintenance costs [10,11,12,13].

Two groups of silicon PUFs are usually distinguished in the literature on the basis of the circuitry utilized to exploit intrinsic variability in the Integrated Circuit (IC) manufacturing process: memory-based and delay-based PUFs. Memory-based PUFs (SRAM [14], DRAM [15,16]), rely on the erratic start-up values of memory cells when the circuit is turned on, while delay-based PUFs (Ring Oscillators (ROs) [17,18,19,20,21,22,23,24,25,26,27], Arbiter [28], Butterfly [29]), take advantage of the differences in delays in signal transmission through two ideally identical paths of an electronic circuit. The memory available in many programmable devices is typically initialized to a fixed value after startup, making SRAM-based PUFs impractical for these devices. On the other hand, arbiter PUFs impose strict layout constraints in order to obtain symmetric delay paths, which is challenging to achieve on programmable devices. As a consequence, FPGA implementations have focused mainly on RO-PUF, where the characteristics of different types of programmable devices can be fully exploited.

FPGAs and SoCs have also played a significant role in the development of TRNGs, e.g., refs. [30,31,32]. Based on the literature, it is possible to again highlight the benefits of programmable devices that exploit mainly three sources of entropy: noise [33,34,35], chaos [36,37,38], and jitter [39,40,41]. As indicated in [42], TRNGs based on jitter are typically easier to integrate and are distinguished by having portable implementations.

Combining the potential of current programmable devices and the benefits of including PUFs and TRNGs as security primitives of a hardware RoT for IoT devices, this work presents a design based on ROs that offers this double functionality, having the ability to act both as a generator of identifiers linked to the devices in which it is implemented or as a source of entropy for the generation of random bit streams. The RO-PUF/TRNG design optimizes the use of the logic resources available in Xilinx Series-7 and Zynq-7000 programmable devices, exploiting their manufacturing features to achieve an optimal bit-per-area rate and providing a high bit-per-time rate. The design has been packaged as a configurable Intellectual Property (IP) module, providing it with an Advanced Extensible Interface (AXI) to facilitate its integration into embedded systems using either a soft- or hard-core general-purpose processor.

The capability of the proposed PUF/TRNG module to produce implementation device-dependent identifiers should be subjected to a rigorous set of tests using specific metrics to assess its dependability and robustness for various configuration options. Likewise, it is also necessary to exhaustively analyze the degree of randomness of the generated bit streams to validate, in accordance with current standards and recommendations, the performance of the proposal as a source of entropy. As with any kind of electronic implementation, the physical characteristics of the device may change over time due to environmental factors such as temperature, voltage, and/or aging, which can affect the design performance. Therefore, it is necessary to provide the RO-PUF/TRNG design with a self-assessment system in order to test and guarantee its performance by monitoring the respective metrics of both of its functionalities.

In relation to this topic, the work also provides a cutting-edge Software Development Kit (SDK) that allows evaluating the levels of reliability, uniqueness, and entropy of PUF/TRNG responses throughout different stages of the product lifecycle: initial development, implementation validation, and field operation. The routines and applications included in the SDK offer a user-friendly automated testing environment that accelerates the process and minimizes the risk of human error by reducing the need for intervention. The ability to compare data between several devices and check the PUF response using different instances in the same device made it possible to thoroughly test the performance of the proposal PUF/TRNG module on a series of test systems specifically designed for this purpose. Additionally, the SDK functions allow regular testing of the security primitive to ensure that the data derived from the inherent physical features are not being compromised, and they are available to be executed at regular intervals or in response to certain events, such as power-on, reboot, or suspected tampering.

To sum up, the main contributions of this work are as follows:

- Design of a security primitive with dual PUF/TRNG functionality, efficient in terms of resource consumption and speed of operation, and provided with a standard AXI4 interface for easy integration in embedded systems implemented on Xilinx Series-7 and Zynq-7000 devices.
- Development of a set of functions and applications included in an SDK to automate RO-PUF/TRNG design testing procedures in order to optimize the characterization and operation processes.
- Performance of a rigorous testing procedure to assess the reliability and stability of the proposed design while acting as a PUF, and to validate the randomness of the generated bit streams while operating as a TRNG.
- Proposal of a dynamic self-assessment mechanism based on the SDK to monitor the RO-PUF/TRNG behavior over time while considering external elements that could have an impact on its performance in both functionalities.

To guide the reader through this document, Section 2 presents the description of the proposed RO-PUF/TRNG design and its integration into the implemented test systems. Section 3 provides information on the SDK created to obtain the metrics used to carry out characterization tasks and to evaluate the PUF/TRNG quality indices. Details of both the metrics and the comprehensive set of tests used to characterize the design and to evaluate the ID generator and entropy source capabilities of the PUF/TRNG module are covered in Section 4, Section 5 and Section 6, respectively. Finally, Section 7 summarizes the main conclusions drawn from the findings of this work.

## 2. RO-Based PUF/TRNG Hardware Design

### 2.1. Overall System Description

The proposed RO-PUF/TRNG design operates by making use of looped structured chains (rings) composed of an odd number of inverters that produce oscillating signals with unique frequencies. All the rings are composed of an equal and even number of NOT gates and a NAND gate that responds to an enable signal to toggle between closing and opening the feedback loop. Each inverter produces an oscillating signal at its output when the loop is closed, the frequency of this signal being primarily determined by the routing between the gates and the multiple delays that accumulate among them. Therefore, it would be expected that ROs described with an equal number of stages and homogeneous layouts could produce output oscillation frequencies with identical features, but even so, the variability inherent in the manufacturing processes of ICs prevents the frequencies from being equal.

Since the initial proposal in [17], different alternatives have been proposed to improve RO-PUF performance from a double perspective of quality and efficiency. The use of specific layout strategies (in ASICs) or placement directives (in FPGAs) to control the spatial arrangement of the ROs, enable signals to minimize mutual influence between system components, and techniques to select the most appropriate challenge-response set are some of the procedures most frequently used to achieve the first objective. Regarding efficiency, this must be considered both in spatial terms, to supply an output bit rate per area large enough to provide secure identifiers of adequate length, and in temporal terms, so that the tasks that the PUF must perform in its enrollment and operation phases are carried out in a reasonable time.

Configurable ROs, which take advantage of the capacity of the Look-Up Tables (LUTs) available in many FPGA devices to implement logic functions that allow selecting different delay paths in each of the RO stages, have been used in different works to improve both the reliability [18] and the efficiency [23] of RO-PUFs. The strategy presented in [25,26] and later extended in [27,43] allows a simultaneous increase in area efficiency and reduction of response time of the system by using as the output of the PUF not only the bit corresponding to the sign of the comparison of the frequencies of the ROs, but also some of the bits corresponding to the difference of values between the two counters used to make the comparison.

The RO-PUF/TRNG design, whose components and operation are described in the following, combines some of the strategies and techniques described in the literature and includes new proposals to increase the functionality and improve the performance in order to adapt its response to the requirements of the currently posed security challenges. Its main features are as follows:

- **Compactness**: the proposed architecture provides a good trade-off between the size of the PUF/TRNG output and the resources it consumes in the programmable device.
- **Configurability**: before performing the synthesis and implementation process, the designer can define the size and location of the RO bank and the length of the counters, as well as select that the implementation conforms to a ‘characterization mode’ or to the normal ‘operation mode’ of the system.
- **Flexibility**: once implemented, the module can be used both as an entropy source and for the generation of hardware identifiers over time to implement a classical fuzzy commitment scheme. Additionally, several configuration options can be explored: the relative location of the two ROs involved in each comparison; the use of Gray or binary code counters; and the specific bits of the counters to include in the PUF output when it is used for ID generation purposes.
- **Quality**: the addition of a challenge selection mechanism to discard the comparisons that most negatively affect the repeatability of the PUF response allows considerably improving its reliability without compromising its uniqueness.
- **Reusability**: the use of a standard connection interface ensures easy integration with different soft- and hard-core processing systems to build SoCs.

**Figure 1:**
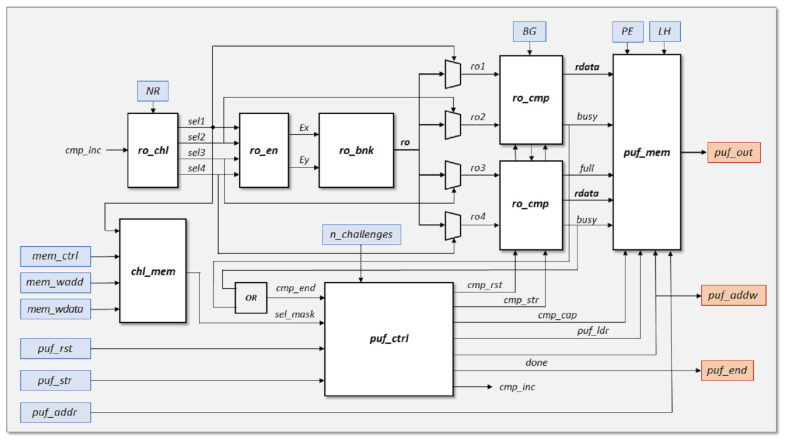
Block diagram of the proposed RO-PUF/TRNG design (blue and red boxes represent inputs and outputs, respectively).

Figure 1 shows the internal organization of the RO-PUF/TRNG design. Similarly to other PUFs based on ring oscillators [17,18,19,20,21,22,24,25,26,27], it operates primarily by comparing the oscillation frequencies of pairs of elements chosen from an available bank of ROs (ro_bnk). In this process, two counters connected to the output signals of the RO-pairs under comparison are used. The counting process is stopped when one of them overflows in order to identify the faster counter, which defines the sign bit, and the value of the slowest counter, from which the bits used to complete the PUF output corresponding to this comparison are captured. The output of the system is a bit stream that is made up of the concatenation of the chosen bits from each of the comparisons after the complete challenge sequence has been applied.

In order to double the bit generation rate in the PUF response, our proposal takes advantage of the two distinct behaviors identified in [27] to perform two comparisons in parallel. In one of them, the comparison is made between two ROs implemented in LUTs placed in the same position of different configurable logic blocks (CLBs), while in the other it is carried out between ROs implemented in LUTs placed in different positions within the same or a different CLB. Furthermore, to improve the reliability of the PUF output, a selection mask created during a prior enrollment process and saved in the challenge mask memory block (chl_mem) can be used to choose the pairs of ROs that will participate in the comparisons during normal PUF operation. The challenge generation (ro_chl) and enable (ro_en) blocks give, respectively, the selection and enable signals for the two pairs of ROs to be compared in each comparison cycle. On the other hand, the PUF output block (puf_mem) receives its information from the two comparison blocks (ro_cmp). For each of the two mentioned comparisons, this block selects the best bits depending on the functionality requested to the module: ID generator or entropy source. In the first case, the sign bit plus a bit of the slower RO counter that offers adequate values of stability (S), probability (P), and entropy (Hintra and Hinter) are taken from one of the comparisons, while two bits of the slower counter satisfying the same requirements are taken from the other one. The bits that present the highest entropy values and probability values closer to 50% are, on the other hand, the most appropriate when the system acts as TRNG. The meaning and calculation of these metrics are detailed in Section 4.

The PUF or TRNG output is finally structured in registers of 32- or 64-bits and stored in an internal memory (*puf_mem*), which may be accessed via the AXI4 interface. This output bit stream is made up of the concatenation of the bits chosen when completing the challenge sequence. The following subsections cover the specifics of how each of the blocks building the design is implemented.

### 2.2. Building Blocks

#### 2.2.1. RO Bank (*ro _bnk*)

**Figure 2:**
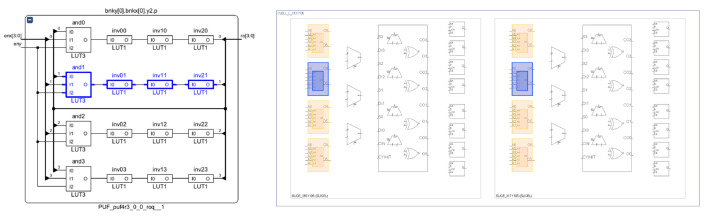
Four 4-stage ROs implemented on a CLB: schematic (**left**) and device representation (**right**), highlighting one of the ring oscillators.

A matrix of CLBs with Nx columns and Ny rows, each of which implements four 4-stage ROs, constitutes the main block of the RO-PUF. Each RO is described with three stages of NOT logic gates, the fourth stage being a NAND gate that serves the dual purposes of receiving column and row enable inputs and closing the feedback loop of the RO, as shown in the diagram on the left side of Figure 2. Each of the eight LUTs in the Xilinx Series-7 and Zynq-7000 CLBs can implement two separate Boolean functions with no more than five inputs [44]. Consequently, it is possible to locate the four ROs within a single CLB and fully utilize the logical resources of the programmable device by employing the proper placement directives in the HDL description.

Location directives are also included in the HDL description of the ROs to force a horizontal layout (shown on the right side of Figure 2) with the goal of making the oscillation frequencies of the ROs as similar as possible. Finally, to ensure that the relative distance between CLBs remains consistent across RO pairwise comparisons while the challenge generation process is in progress, the same procedure is used to make the RO placement scheme within the RO bank follow a snake pattern.

#### 2.2.2. Challenge Generator (*ro _chl*)

The challenge sequence supplied by the challenge generator block chooses the two RO pairs to be compared in each comparison cycle. Any two RO pairs can be compared, even those sharing a single CLB. The block has four outputs (sel1−sel4) that are routed to the block that generates the enable signal, as well as to the multiplexer control inputs used to select the ROs that supply the clock inputs to the comparison blocks. A counter that increases by one with each comparison cycle produces the sel1 signal values. The other selection signals are calculated as a function of sel1 as shown in Equation (1),

(1) sel2=sel1+1+Δ×4;sel3=sel1+2;sel4=sel1+6+Δ×4

where Δ enables us to specify the separation between ROs in terms of the amount of CLBs. sel1 and sel2 choose ROs that are implemented in LUTs placed in different locations inside the same or contiguous CLBs (if Δ=0) or in two different CLBs (for Δ in the interval [1,Nx×Ny−1]). On the other hand, the elements involved in the other simultaneous comparison, controlled by sel3 and sel4, correspond to ROs implemented in LUTs situated at the same position of two CLBs that can be contiguous (Δ=0) or separated by a specific distance (Δ≠0).

The proposed RO-PUF/TRNG offers a run-time option to choose whether the two simultaneous comparisons are made between the closest or farthest ROs of each type inside the RO bank, giving users the flexibility to select between two configurations. In the first situation, a null value is set for Δ based on the **NR** (Nearby/Remote) option setting, whereas in the second case, an internally determined value is used based on the Nx and Ny implementation parameters that determine the size of the RO bank.

#### 2.2.3. Challenge Mask Memory (*chl _mem*)

One of the main novelties of our proposal is the inclusion of a challenge selection mechanism that allows discarding those comparisons of pairs of ROs that compromise the reliability of the PUF to a greater extent. To speed up the operation of the module, once the pairs to be discarded have been identified in an enrollment process, the generated selection mask can be stored in the memory provided by this block. Subsequently, at the beginning of each comparison cycle during the PUF invocation, the signal sel1 will be decoded to identify the bit corresponding to the comparison in progress, which is analyzed by the control block to determine whether the comparison process starts or returns to activate the cmp_inc signal to discard (in just a few clock cycles) this comparison and move on to the next pair of ROs.

#### 2.2.4. Enable-Signals Generator (*ro _en*)

Only the four ROs selected by the current challenge are enabled in each comparison cycle with the aim of limiting the activity of the RO bank components to reduce energy consumption and eliminate mutual impacts among them. The row (Ey) and column (Ex) enable signals are activated by the enable signal generation block (ro_en) to close the feedback loop of the four ROs determined by sel1 through sel4. The values of the Nx parameter are limited to powers of two in the PUF design to make the implementation of this block simpler.

#### 2.2.5. Comparison Block (*ro _cmp*)

In order to make the two parallel comparisons that produce the response corresponding to a challenge, the PUF contains two identical comparison blocks (ro_cmp). Each of these blocks includes two Gray-code counters built from binary counters and using the output from the two chosen ROs as count signals. The design includes the logic needed to stop the functioning of the other counter when one of them has reached overflow. The size of the counters is determined by a parameter selected during the design synthesis and implementation processes.

End-of-count comparison is always performed with Gray-code counters to ensure that the result is independent of delay differences in the corresponding binary counter bits. However, the bits that make up the output of the system, whether it acts as a PUF or as a TRNG, can be taken from both types of counter depending on the value assigned to the **BG** (Binary/Grey) option, which provides two possible alternatives that, together with other design configuration parameters, allow different reliability-safety trade-offs to be established.

When the PUF control block activates the cmp_str signal, the comparison cycle begins simultaneously in both blocks and stops when the busy signals of both blocks drop to 0, indicating that a counter has reached its highest possible number. The inputs of the last stage in the block diagram of the design can then access the signal that identifies the fastest counter in one of the blocks and the values of the slower counters in both blocks.

#### 2.2.6. PUF Output Block (*puf _mem*)

The output stage (puf_mem) has two distinct functions. On the one hand, it chooses the bits that will be included in the system response for each challenge. On the other hand, when the application of the series of challenges advances, it is responsible for structuring the subsequent responses in 32- or 64-bit registers and storing them. The PUF/TRNG output will be read from this memory after its operation is finished.

The specific bits that will contribute to the output depend on the operation specified for the module using the **PE** (PUF/Entropy Source) run-time configuration option. The bits selected for the PUF operation are determined by the type of ROs being compared as well as by the **LH** (Lower/Higher) option defined by the user. As in other works in the literature [25,26,27], to make them independent of the size of the counters, in the text, the sign bit is denoted as bit 0 and the rest of the bits are named in ascending order, bit 1 being the MSB of the counter value. According to this notation, bit 6 and 7 (for the Lower option) or bits 7 and 8 (for the Higher option), both from the slower counter, are chosen to form a part of the PUF output for comparisons between ROs implemented in LUTs placed in different locations of the CLBs. The sign bit in association with bit 7 (Lower option) or bit 8 (Higher option) of the slower counter contribute to the output of the PUF in comparisons between ROs implemented in LUTs placed in the same location of different CLBs.

For TRNG operation, the two least significant bits of the slower Gray-code or binary counters are selected. The choice of these bits for each case will be justified later when discussing the results of the design characterization tests carried out in the early stages of its development.

The four selected bits in each comparison cycle are sent to a shift register (the size of which is a function of the width of the interconnect interface selected when synthesizing the module) in charge of organizing the output bit stream and storing it in consecutive locations of the PUF memory, which is implemented using Block RAM (BRAM) in the programmable device. The address and data buses connected to this memory can be used to access the PUF output from outside the design.

**Figure 3:**
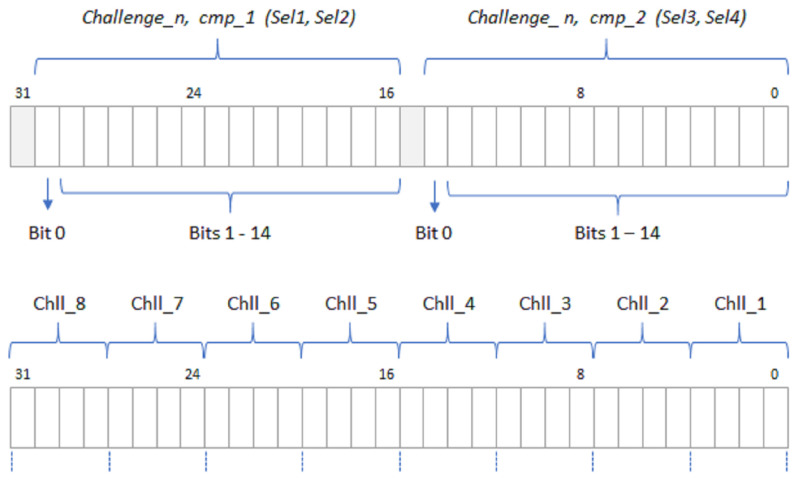
PUF/TRNG output register in characterization (**upper**) and operation (**lower**) mode when using 14-bit counters and a 32-bit interconnect interface.

In addition to its normal ‘operation mode’, the design can be synthesized in an alternative ‘characterization mode’ which allows the collection of all counters output bits in each comparison cycle, making it easier to analyze the system behavior to improve its performance or compatibility with other devices. In this case, the data corresponding to the two comparisons performed in each comparison cycle are stored in a memory address. Both modes use the same mechanism to provide the module response, differing only in the number of registers in the output memory (Figure 3).

#### 2.2.7. Control Block (*puf _ctrl*)

The puf_ctrl block provides the control signals required to sequentially operate the blocks building the proposed design. A Finite State Machine (FSM), which produces the signals to control the cycles of RO-pair comparisons, and a sequence of processes to produce the signals defining the successive operation stages and controlling access to the PUF memory, are both included in the HDL description within this block.

The FSM receives two internally generated signals: sel_mask, which determines whether the RO pairs involved in the current operation cycle will be discarded as a result of a previous enrollment process, and cmp_end, which indicates the end of the two comparisons. The FSM also receives two external inputs, puf_str, which sets the start of the PUF operation, and n_challenges, which specifies the number of challenges used in the PUF invocation. The signals cmp_rst and cmp_start, which are used to initialize and begin the comparisons, respectively, as well as the cmp_cap signal, which is used to capture the bits chosen in the two simultaneous comparisons, are provided as output.

**Figure 4:**
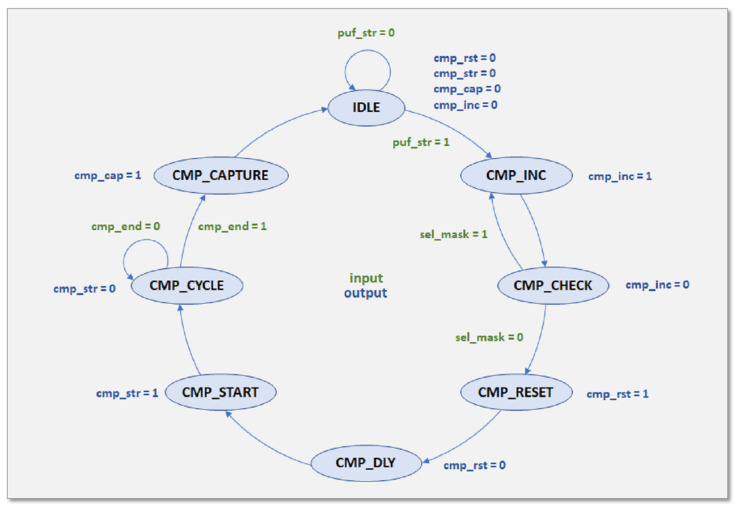
State diagram of the FSM included in the control block.

The FSM state diagram is shown in Figure 4. The four output signals (cmp_rst, cmp_str, cmp_cap, and cmp_inc) are disabled by being set to 0 when the FSM process starts in the IDLE state. The FSM enters the CMP_INC state when the puf_str signal is high, causing the cmp_inc signal to be activated to increase the counter utilized in the challenges-generating block to set the select signals of the RO pairs included in the two parallel comparisons. In the following clock cycle, the FSM goes to the CMP_CHECK state, where the signal cmp_inc is disabled and the sel_mask flag from the challenge memory block is assessed to determine if the FSM advances to the CMP_RESET state or comes back to CMP_INC. The FSM enters the CMP_RESET state when a non-discarded comparison is found, at which point cmp_rst is activated to reset the counters for the two comparison blocks. In the next cycle, the FSM enters the CMP_DLY state and deactivates cmp_rst. Then, after another clock cycle, it enters the CMP_START state and activates cmp_str to begin the execution of both comparison blocks. The FSM remains in the CMP CYCLE state until the cmp_end input is set and both comparisons have finished. Then, it enters the CMP_CAPTURE state and turns on cmp_cap to capture the four-bits (or thirty-two bits for characterization mode) that are transferred to the shift register to be stored in the PUF memory. The FSM returns to the IDLE state again in the following clock cycle, waiting for the beginning of a new comparison cycle.

The number of applied challenges is tracked by a counter that is increased each time the cmp_cap signal is high, which implies the conclusion of a comparison cycle. The puf_ldr signal is activated to store the content of the shift register in the PUF memory location indicated by puf_wa after eight or sixteen challenges (for 32- and 64-bit registers, respectively) have been successfully completed (one or two cycles when the PUF is implemented in characterization mode). The value of the signal is then increased by one. Finally, once the number of challenges evaluated reaches the value specified by the n_challenges input, the done output signal is activated to indicate that the PUF call has been completed.

### 2.3. Core Implementation and Performance Evaluation

The design detailed in the previous section was synthesized and implemented from VHDL descriptions using the tools offered by the Xilinx Vivado Design Suit. Throughout the experimental evaluation, different programmable devices from the Series-7 and Zynq-7000 families were considered in order to estimate their performance in terms of resource occupation and response time as a function of the parameters that determine the size of the PUF/TRNG core.

Table 1 shows the utilization of resources in terms of LUTs, registers, slices, and block RAMs (and occupation percentage in brackets) for four core implementations in Spartan-7, Artix-7, Zynq-7000, and Kintex-7 devices available in Arty S7, Nexys A7, Pynq Z2, and Genesys 2 development boards, respectively. The occupied resources can vary slightly depending on the choices selected for the synthesis and implementation design tools.

**Figure 5:**
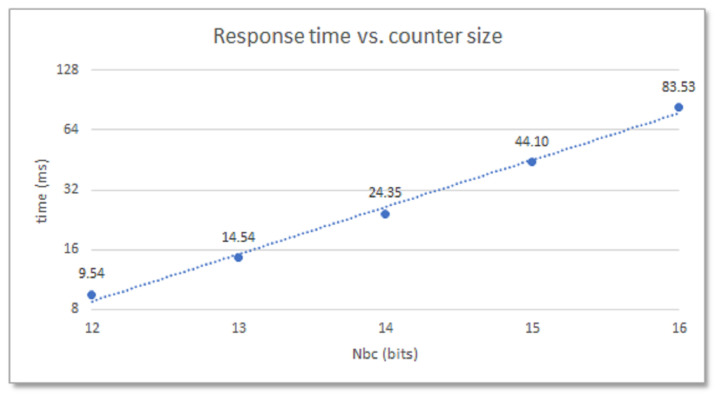
Semi-logarithmic representation of the response time versus counter size for a 480-RO PUF on a Pynq Z2 development board.

The specifications of the programmable device, such as family, part, and speed grade, with which the module is implemented (which determine the oscillation frequency of the ROs), as well as the parameters used when implementing the design, mainly those that define the size of the RO bank and the length of the counters (which determine the number of comparisons to be performed) have a major impact on how fast the system operates. This behavior is shown in Figure 5, which illustrates how the response times of a 480-RO PUF implemented in the Zynq-7000 device of a Pynq Z2 board change when the size of the counters moves in the range 12- to 16-bits. With an average oscillation frequency of the ROs on this device around 315 MHz, the PUF takes almost 25 ms to produce its output when implementing 14-bit counters, but only slightly more than half of this time when the size of the counter is decreased to 13-bits.

### 2.4. IP Encapsulation and Test System Integration

To facilitate its incorporation into the RoTs of embedded systems as a fundamental building block for the derivation of identifiers and random bit sequences, the RO-PUF/TRNG core was encapsulated as a configurable IP module and provided with a standard interface for connecting with hard- or soft-core processors. The AXI4-Lite protocol, suitable for connecting general-purpose processors with low- or medium-speed memory-mapped peripherals, was selected to achieve low-resource implementation.

**Figure 6:**
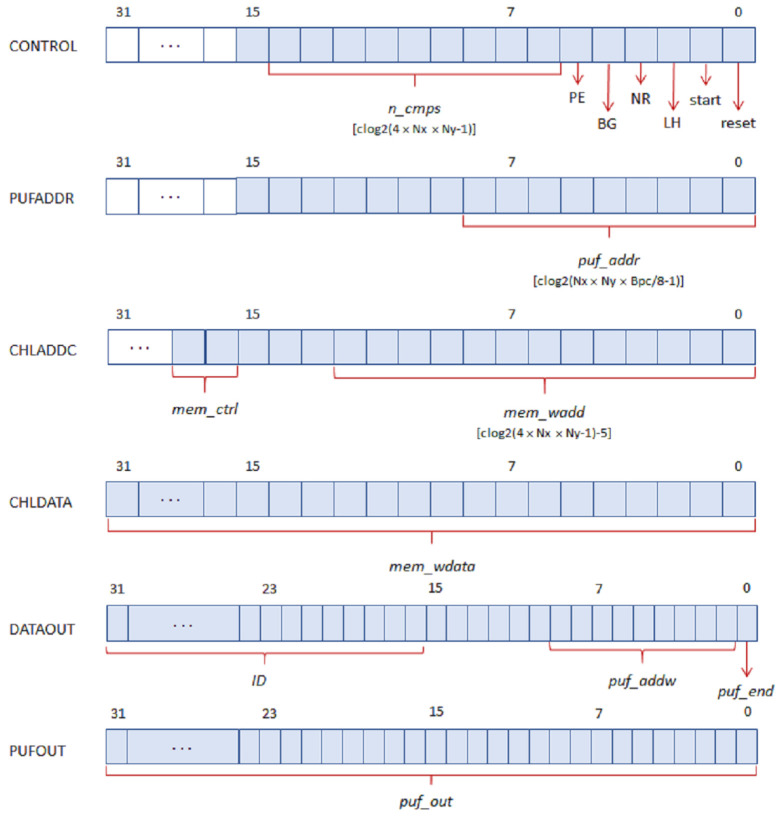
Input and output IP module registers.

The inputs and outputs represented in Figure 1 in blue and red, respectively, are connected to six I/O registers following the bit association scheme illustrated in Figure 6 for the case where a 32-bit interface is used.

The PUF/TRNG receives the initialization (puf_rst) and operation start (puf_str) signals, as well as the number of challenges (n_challenges) and configuration choices (PE, BG, LH, and NR) through the CONTROL input register. With the exception of the first field, which is dependent on the RO bank size established when implementing the PUF/TRNG, all fields have constant lengths. After the module has completed its operation, PUFADDR is utilized as an input register to access PUF memory. As the design is synthesized, the maximum number of bits to represent the read memory addresses (puf_addr) is appropriately calculated as a function of the length of the PUF response and the number of memory cells required to store it. The other two input registers are used to store the challenge selection mask for the PUF. CHLADDC provides control and address signals to determine the write or read of the internal memory, as well as to select the address of the cell accessed in the operation. The maximum number of bits to represent the write-memory address (mem_wadd) is automatically adjusted as a function of the PUF size. Finally, the content of the challenge selection mask is provided through the mem_wdata field in the CHLDATA register, whose size coincides with the register length chosen when synthesizing the system.

There are three fields in the output register named DATAOUT. When the PUF/TRNG IP is instantiated in a design, a user-defined identifier can be set for debugging or verification purposes, which is accessible through the ID field of this register. puf_addw represents the address of the last memory position that contains the PUF output, enabling the user to confirm that it has the correct length. puf_end is a signal to indicate the PUF has completed its work. Finally, the field puf_out in the PUFOUT register allows accessing the content of the output memory location referenced by puf_addr. When using a 64-bit AXI interface, the size of the fields mem_wdata and puf_out is doubled, so the number of read and write operations over the bus required to set the challenge selection mask and obtain the IP output can be reduced by half.

**Figure 7:**
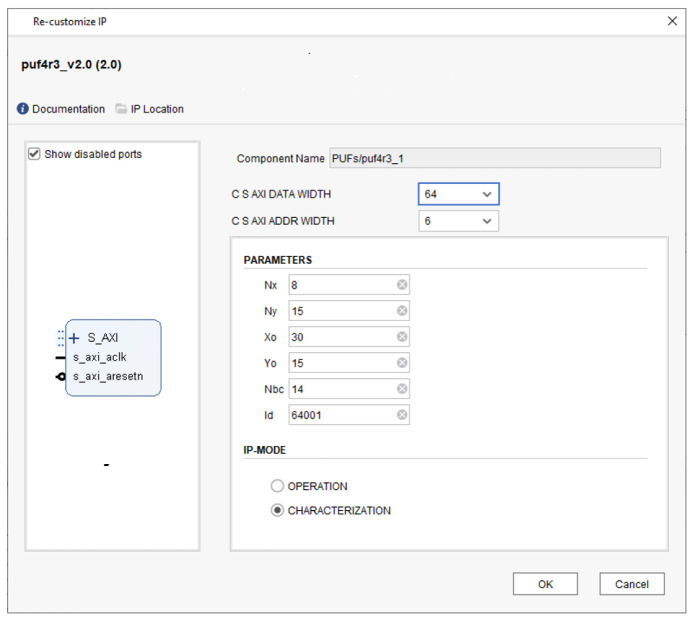
PUF/TRNG IP module Graphical User Interface.

To customize the design implementation and enable its use in a wide range of applications, it has been significantly parameterized. When using Xilinx’s Vivado IP Integrator tool to include the PUF/TRNG IP into their own system, designers can set some of these parameters by means of the Graphical User Interface (GUI) shown in Figure 7. It illustrates the set of parameters that can be set through the GUI, which includes the number of rows (Ny) and columns (Nx) of contiguous CLBs that constitute the RO bank, its location within the programmable device (Xo, Yo coordinates), the length of the counters used to compare the ROs frequencies (Nbc), and the identifier associated with the PUF (ID). The operation mode and the use of 32- or 64-bits for AXI interface and internal memories are also be defined in the GUI.

#### 2.4.1. Test Systems

The metrics commonly used to evaluate the quality of PUF and TRNG proposals require the processing of a large amount of output data obtained from a relatively large number of implementations on different devices (or in different locations of the same programmable device). For this reason, with the idea of accelerating the validation stage of the proposed solutions, different instances of the PUF/TRNG IP were incorporated into different HW/SW hybrid test systems implemented on development boards with different Xilinx Series-7 FPGAs and SoCs. The processors of these integrated systems are used both to access the module through a set of high-level language controllers and to carry out the characterization and evaluation processes on-line when it acts as ID generator or source of entropy. In the first case, the quality of the PUF is evaluated using the set of conventional metrics that determine its reliability and uniqueness. In the second case, the validation of the bit sequences provided by the TRNG is carried out with the tests and recommendations proposed by the National Institute of Standards and Technology (NIST) [45,46].

**Figure 8:**
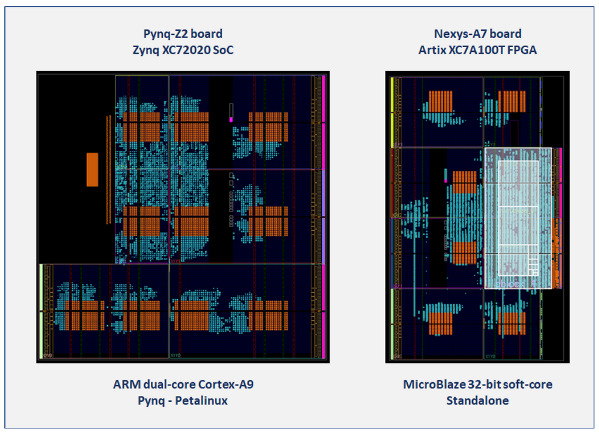
Device view of test system implementations in Pynq Z2 (**left**) and Nexys A7 (**right**) development boards.

For both small series development and deployment, as well as validation and performance analysis of new designs, programmable SoCs, which integrate a Processing System (PS) and Programmable Logic (PL) within the same IC, have proven to be very suitable platforms by combining the adaptability of the software and the efficiency of implementing a part of the system in specialized hardware designed for a particular use. Exploiting these features, a test system was built using the Xilinx Zynq-7000 SoC device provided on the Pynq Z2 development board, upon which a series of C-coded routines was executed on one of the available ARM cores to perform the initial characterization and validation of the proposed PUF/TRNG design. This test system, which appears on the left side of Figure 8, instantiates ten identical PUF/TRNG IP modules, each of which has a counter size of 14-bits and eight rows and 15 columns of CLBs (480 ROs). Different clock zones of the device were used to locate the distinct RO banks. The implementation tools placed the remaining parts of each PUF in nearby resources in the same clock zone as the RO bank.

On the other hand, to build the experimental setup used to analyze the behavior of the module under changes in operating conditions (temperature and supply voltage), a second set of test systems was implemented on the Artix-7 FPGA of the Nexys A7 development board using a 32-bit MicroBlaze processor to run verification and test programs in a standalone environment. As shown on the right side of Figure 8, the resources available in this device allowed it to accommodate six copies of the IP module with 480 ROs and 14-bit counters. In both cases, orange cells correspond to the RO banks whose positions were fixed when the PUFs were instantiated, white boxes mark the zones defined by the ‘pblock’ directives to place the components of the MicroBlaze processing system, and the cells in green show fully or partially used device resources.

Table 2 summarizes the resources needed to implement the different test systems used to perform the measurement and verification tasks addressed in this work. As can be seen, despite instantiating a lower number of IPs, the test systems implemented on the Nexys A7 development board use a greater number of resources (LUTs, registers and BRAMs) as a consequence of the inclusion of the MicroBlaze soft-core and the external-memory controller required to support the evaluation and monitoring software.

## 3. Software Support for Characterization and Performance Evaluation

As mentioned above, the proposed PUF/TRNG was envisioned as a configurable IP module that can be incorporated into an embedded system and connected to a general-purpose processor to provide the RoT for security applications. For this reason, with the aim of facilitating its use from high-level programming languages, as well as to speed up the characterization and performance evaluation of the designs implemented in the test systems throughout the development phases and to monitor its behavior during the operation phase, a software development kit was generated that includes drivers, functions, utilities, and applications necessary to control and evaluate the operation of the IP module.

The SDK was initially developed using the Python Productivity for Zynq (PYNQ) framework available for the Pynq Z2 board [47]. This environment provides a Python framework on an embedded Linux operating system that facilitates the interaction between hardware and software components of an embedded system and the development of applications. For efficiency reasons, the C-API available at [48] was used as an alternative to the Python framework, because it provides the same functionality as the PYNQ environment through a set of C-routines that are compiled to generate executable code. To extend its use for test systems implemented on other devices and development boards, the SDK was later updated so that it could also be used on standalone systems and other Linux distributions that do not include the PYNQ environment. The current version of the SDK provides support for the different test systems and/or operating environments (standalone, Pynq on ARM, Petalinux on MicroBlaze) used to obtain the results gathered in the next sections. It is easy to install and use and integrates under a single scheme a set of low- and high-level routines that simplify the usage and evaluation of the PUF/TRNG module for both its operation as an ID generator and as a random number generator.

The basic functions to control the operation of the IP module are summarized in Table 3. The first three functions use low-level drivers to establish the interface between hardware and software and perform the two basic tasks for the operation of the system as an ID generator or source of entropy: application of challenges and reading of results. The other three functions perform higher-level tasks related to the challenge selection mechanism and obtaining metrics to assess the quality of the IP module when it acts as an ID generator.

### Application Programs

Using the functions included in Table 3, together with a set of routines for calculating and presenting the required metrics, different high-level applications were added to the SDK to quickly and easily carry out characterization and evaluation tasks in the different phases of development of the IP module, as well as to facilitate its monitoring once integrated and in operation on an embedded system. The functionality and objective of these applications are outlined below, although, to facilitate their understanding, the calculation and meaning of the metrics used in each case will be detailed in the following sections, when analyzing the results obtained through their execution in the implemented test systems.

***puf_getdata*** was the most widely used application in the early stages of development of the PUF/TRNG module. It runs successive series of tests for each of the PUFs included in a test system in order to verify their correct operation and capture data for off-line evaluation of their characteristics by means of C-coded programs or Matlab scripts. On the other hand, the on-line execution of this task by the embedded system itself is supported by ***puf_bitselect***, which runs a series of tests for each of the PUFs in a test system and extracts the metrics that allow selecting (characterization mode) or analyzing (operation mode) the bits of the counters that form the PUF output. The metrics calculated by this application will be detailed in Section 4, where the strategy followed to select the most appropriate bits for the two functionalities of the IP module is described.

The challenge selection mechanism proposed in the work can be explored using ***puf_enrollment***. This application program executes the enrollment processes for the PUFs incorporated in a test system to obtain their reference outputs. Optionally, a selection mask indicating the challenges (pairs of ROs) with the worst responses from the stability point of view can also be generated during each enrollment stage, so that they can be eliminated from the PUF output in order to improve its reliability.

The beneficial effects of the application of this selection mechanism on the quality of the IP module, when used as an ID generator, can be evaluated by means of ***puf_HDintra*** and ***puf_HDinter***. The first one evaluates, for each of the PUFs instantiated in a test system, the Hamming distance with respect to its reference output (HDintra) for successive runs, while the second evaluates the Hamming distance with respect to the other PUFs (HDinter) for successive runs. To do this, in both cases, an enrollment process is performed for each PUF to obtain the reference output and the challenge selection mask that indicates the discarded comparisons.

Parameters that determine the quality of the IP module when used to obfuscate and recover a secret key can be easily evaluated by the ***puf_reliability*** and ***puf_uniqueness*** application programs, which evaluate the reliability and uniqueness of the PUFs implemented in a test system, respectively. In both cases, an enrollment process is first performed for each PUF to obtain its reference output. Subsequently, the key masks obtained by applying an Error Correction Code (ECC) with a given repetition factor to the responses of successive series of invocations to the PUF are analyzed.

Once in the system operation phase, the quality of the module as an ID generator can be periodically monitored with the help of the ***puf_test*** application. This command accepts as input parameters the length of the key and the repetition factor of the ECC, and performs the following tasks: (1) processes the input parameters to calculate the number of challenges that can be discarded; (2) executes an enrolment process to obtain the PUF reference output and the challenge selection mask; (3) evaluates the HDintra metric after applying the challenge selection strategy; and (4) invokes the PUF repeatedly to verify its reliability as an ID generator using the chosen configuration.

Finally, the SDK also incorporates two applications to measure or validate the quality of the IP module when working as a TRNG. As in the evaluation of the PUF functionality, the TRNG functionality requires a data collection stage for further processing that, for this functionality, can be performed both on-line and off-line. In this case, the ***trng_getdata*** function is responsible for collecting data that meet the formatting requirements of the NIST 800-90b recommendation. Subsequently, the ***trng_validation*** function takes the collected data and processes them to verify that the characterization made to the TRNG in terms of entropy remains within an adequate range of values.

Using these applications, a series of specific tests were generated in order to repeatedly call all the PUFs instantiated in the test systems and process their corresponding output data. When these tests are launched, the user can define the number of challenges, the number of PUF calls, the number of runs (i.e., times the set of tests is repeated), the debug level, and other options. Different strategies can also be applied by combining the configuration options for selection of PUF or entropy source functionality (**PE**), binary or Gray-coded counters (**BG**), nearby or remote ROs (**NR**), and lower or higher bits (**LH**); the latter only when used as ID generator. All the tests can be executed using command-line or shell scripts, and their output data can be captured and saved in files for posterior processing. The files required to program the device, run the applications, and reproduce the tests on the Pynq Z2 board, together with the relevant documentation, are available in the repository IMSE.HwSec (accessed on 13 April 2023).

The objective pursued with the realization of the different tests varied throughout the module development process. The tests carried out in the early stages of development were focused on characterizing the responses of the module with the aim of validating the design building blocks and selecting the most suitable bits for the dual functionality of the IP module. Subsequent tests, however, focused on evaluating the quality of the generated identifiers and sequences of bits and on the analysis of the influence of the proposed solutions and the design configuration options on the metrics that quantify that quality. The following sections detail the tests carried out and discuss the main results obtained.

## 4. PUF/TRNG Characterization and Bit Selection Strategy

To carry out the task of selecting the bits that should constitute the output of the system for the two foreseen functionalities, an extensive battery of tests was executed, using the ***puf_bitselect*** application to obtain on-line the metrics for the different implemented test systems. In all the cases, test systems implemented in characterization mode were used to obtain the set of metrics for all the bits of the counters corresponding to the ROs with the lowest oscillation frequencies in each comparison. The measurements made determine the stability, probability, and entropy of the extracted bits as a function of the parameters used to configure the module. The meaning of each of these metrics is summarized below.

- **Stability (S)** provides a measure of the capacity of the *i*-th bit of the counters to obtain the same value in response to successive invocations of the PUF/TRNG module, thus determining the level of reproducibility of this value. Its ideal value is 1 from the ID generation and 0.5 from the TRNG perspective. The stability of bit *i* is calculated as the average of the stability associated with this bit in the *n* comparisons made to obtain the PUF/TRNG output (Equation (2)), which is in turn calculated based on the probability that the bit is 1 or 0 after a number of PUF invocations (Equation (3)).
  (2) $$S_{i}=\frac{1}{n}\sum_{j=1}^{n}s_{i,j}\left(RO_{j}\right)\;$$
  (3) $$s_{i,j}\left(RO_{j}\right)=\{\begin{array}{cc} p_{j}\left(b_{i}=1\right) & if\;p_{j}\left(b_{i}=1\right)\geq 0.5\\ 1-p_{j}\left(b_{i}=1\right) & if\;p_{j}\left(b_{i}=1\right)<0.5 \end{array}$$
- **Probability (P)** represents the feasibility of obtaining the value 1 at the *i*-th position of the counters in *n* competitions, which allows the possible bias in the PUF/TRNG output to be analyzed. Its ideal value is 0.5 from both the ID generation and TRNG perspectives. The probability of bit *i* of the counters in the global of the *n* comparisons is calculated as the average of the probability in each of them after successive *t* PUF/TRNG invocations, as shown in Equation (4).
  (4) $$P\left(b_{i}=1\right)=\frac{1}{n}\sum_{j=1}^{n}p_{j}\left(b_{i}=1\right)=\frac{1}{nt}\sum_{j=1}^{n}\sum_{k=1}^{t}b_{i,j,k}=\frac{1}{tn}\sum_{k=1}^{t}\sum_{j=1}^{n}b_{i,j,k}$$
- **Intra entropy (Hintra)** calculates the uncertainty that exists to obtain the value 0 or 1 in the *i*-th bit of the counters in *n* RO-pair competitions. Its ideal value is 1 from both the ID generation and TRNG perspectives. The entropy associated with bit *i* of the counters of a certain PUF is calculated according to Equation (5),
  (5) $$Hintra_{i}=p_{l}\left(0\right)log_{2}\left(p_{l}\left(0\right)\right)+p_{l}\left(1\right)log_{2}\left(p_{l}\left(1\right)\right)$$
  where $p_{l}\left(0\right)$ and $p_{l}\left(1\right)$ correspond, respectively, to the probability of obtaining 0 and 1 in this bit after repeatedly invoking the PUF and taking into account all comparisons, that is, all pairs of ROs. They are calculated according to Equation (6) as the average of the most probable values (the reference values) obtained in each comparison by repeatedly invoking the PUF.
  (6) $$p_{l}\left(1\right)=\frac{1}{n}\sum_{j=1}^{n}round\left(\frac{1}{t}\sum_{k=1}^{t}b_{i,j,k}\right)\;\;\;\;\;\;\;\;\;\;p_{l}\left(0\right)=1-p_{l}\left(1\right)$$
  The above stability, probability, and Hintra values are for a single PUF. Those that usually appear in the tables and figures throughout this text to characterize a design are calculated as the average of the values of *m* different PUFs (implemented on the same or in different programmable devices), as shown in Equation (7).
  (7) $${\bar{S}}_{i}=\frac{1}{m}\sum_{l=1}^{m}S_{i,l}\;\;\;\;\;\;\;\;{\bar{P}}_{i}=\frac{1}{m}\sum_{l=1}^{m}P_{i,l}\;\;\;\;\;\;\;\;\bar{H}intra_{i}=\frac{1}{m}\sum_{l=1}^{m}Hintra_{i,l}$$
- **Inter entropy (Hinter)** calculates the same uncertainty as Hintra but differs in that it calculates the results of competitions of *n* RO pairs located at the same position of different instances of the module. The entropy associated with bit *i* of the counters for *m* PUF implementations is calculated as the average of the entropy in each of the comparisons, according to Equation (8),
  (8) $$\bar{H}inter_{i}=\frac{1}{n}\sum_{j=1}^{n}Hinter_{j}=\frac{1}{n}\sum_{j=1}^{n}p_{j}\left(0\right)log_{2}\left(p_{j}\left(0\right)\right)+p_{j}\left(1\right)log_{2}\left(p_{j}\left(1\right)\right)$$
  where $p_{j}\left(0\right)$ and $p_{j}\left(1\right)$ correspond to the probability of obtaining 0 and 1, respectively, in this bit in comparison *j* after repeatedly invoking the different PUFs. They are calculated by Equation (9) as the average of the most probable values (the reference values) obtained for this bit in this comparison by repeatedly invoking the different PUFs.
  (9) $$p_{j}\left(1\right)=\frac{1}{m}\sum_{l=1}^{m}round\left(\frac{1}{t}\sum_{k1}^{t}b_{i,j,k}\right),\;\;\;\;\;\;\;\;\;\;p\left(0\right)=1-p\left(1\right)$$

**Figure 9:**
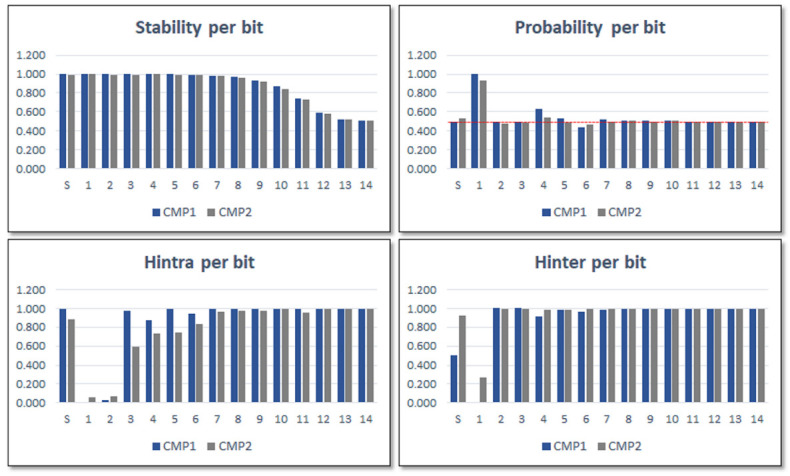
Stability, probability, and entropy metrics calculated for each bit of the counters (average values for 100 combinations of development board, PUF instance, and configuration options). The red line corresponds to the ideal proability value.

To choose the best bits for creating the PUF and TRNG outputs, the stability, probability, and entropy values for each bit of the counters were calculated from data obtained in five Pynq Z2 development boards implementing a test system with ten PUFs in characterization mode. The call to each PUF was executed 1000 times for each of the four configurations defined by **GB** and **RN** options and 480 comparisons (the maximum possible) were performed in each execution. Figure 9 shows the mean values of the results obtained for the 14 bits of the counters plus the sign bit in the two comparison blocks. The results reveal that the stability values decrease in the direction from MSB to LSB, whereas the Hintra values grow in the same direction. The results also show that Hinter for the sign bit only reaches an acceptably high value in the second of the comparisons, as well as that probability values of most of the bits are close to ideal.

From the perspective of ID generation functionality, it was necessary to determine a trade-off to choose the bits to use in the operation mode among those whose metrics show the values closest to their respective ideal values, considering that stability and entropy increase in opposite directions. Accordingly, the most suitable bits to build the PUF output correspond to the sign bit plus one of the bits 7–8, for comparisons between ROs implemented in LUTs placed in the same location of different CLBs (COMP2), and two of the bits 6–8, in the other case (COMP1).

Given that the probability approaches its ideal value and the entropy increases in the same direction as stability decreases, from the perspective of TRNG functionality, the characterization stage will concentrate on determining the number of least significant bits that will be chosen from each of the comparison blocks, as well as the best configuration(s) of the IP module. Sign bits will not be taken into account when the system is implemented in operation mode and utilized as a TRNG since the entropy and stability results for the sign bits are less adequate from a TRNG perspective than those of the Least Significant Bits (LSBs) of the counters. We chose to characterize the two comparison strategies independently utilizing one, two, and four LSBs to carry out the analysis, presented later in Section 6, as the original PUF design extracts four bits in each comparison cycle.

**Figure 10:**
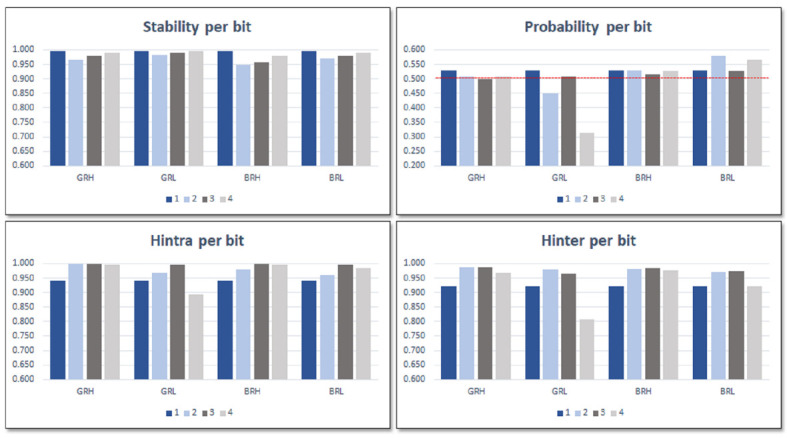
Average stability, probability, and entropy per bit associated with the bits selected for ID generation using different configurations in test systems implemented in operation mode. The red line corresponds to the ideal proability value.

For test systems implemented in operation mode, ***puf_bitselect*** can be used to evaluate the stability, probability, and entropy of the four bits selected based on the functionality and the specific configuration chosen when the PUF/TRNG is invoked. Figure 10 displays the mean values of these metrics after applying $10^{3}$ times a full set of 480 challenges on every one of the 50 PUFs while the IP module acts as an ID generator. The data in each bar graph are organized and labeled into categories that represent the selected bits based on the combinations of the **LH** and **GB** configuration settings and the relative location of the compared ROs. The sign bit of the second comparison is always represented by the bars labeled 1. When comparing RO pairs located in LUTs that are in identical positions in different CLBs, label 2 represents bit 7 (L) or 8 (H) of the comparison results. Label 3 represents bit 6 (L) or 7 (H), and label 4 represents bit 7 (L) or 8 (H), both when comparing ROs located in LUTs that are in different positions regardless of the CLB.

The configuration settings that involve lower bits of the counters (L) exhibit more stability, as shown in the graphs, despite the fact that their probability values deviate from ideal values and their entropy values are lower than those of the configurations that involve higher bits (H). There were no noticeable variations in the four metrics with respect to the relative positions of the compared ROs (nearby or remote), that is, they produce different outputs but with similar characteristics in terms of the metrics considered. For this reason, when analyzing the behavior of the PUF/TRNG module as an ID generator in the next section, we will usually limit the results shown to those corresponding to four of the eight cases that arise from considering the type of counter (B or G), the relative location of the compared ROs (R or N), and the bits that contribute to the output of the PUF (L or H).

## 5. Performance Evaluation of the PUF/TRNG as Id Generator

The purpose of this evaluation assignment is to quantify the **reliability** and **uniqueness**, which are the two key characteristics that define the quality of a PUF. The reliability of the PUF response defines how often it is reproduced upon subsequent device invocations, while uniqueness defines the capacity of the PUF to produce an output that is singular and unambiguously identifies that device. It is possible to quantify both magnitudes for a given PUF by measuring the Hamming distances between the codes resulting from applying the challenge sequence repeatedly to the same PUF (**intra-Hamming distance**, HDintra), and to additional copies of it placed at different locations on the same programmable device, or at the same location on various programmable devices (**inter-Hamming distance**, HDinter), respectively. The ideal HDinter value is 50%. HDintra has a desirable value of 0%, that is, a PUF invocation will always give the same response. However, this value is challenging to achieve due to the various noise sources present in the IC, which typically requires the use of ECCs and extends the bit stream size of the PUFs. The intra-Hamming distance is estimated as:

(10) $$HDintra=\frac{1}{m\times t}\sum_{i=1}^{m}\sum_{j=1}^{t}HD\left(R_{r},R_{i,j}\right)\times 100\%$$

where *m* is the number of implementations of the same PUF on different development boards (i.e., different devices), *t* is the number of times the functionality of each PUF is invoked, HD is the Hamming distance, and $R_{r}$ is the reference response calculated in an enrollment process as the mode over all the PUF responses.

The inter-Hamming distance is calculated by:

(11) HDinter=1m2∑i=1m−1∑j=i+1mHD(Rri,Rrj)×100%

where *m* is in this case the total number of PUF implementations, calculated as the product of the number of PUFs included in the test system by the number of devices in which it is implemented.

The results obtained when the PUF behavior is evaluated by means of ***puf_HDinter*** and ***puf_HDintra*** considering different configuration options are summarized in Table 4. They correspond to the test systems implemented on 5 Pynq Z2 development boards including ten instances of the PUF/TRNG IP module with 32-bit AXI4-Lite interface. All modules use 14-bit counters and incorporate a bank of 480 ROs, capable of providing a different 1920-bit output for each of the eight possible configurations. Column 1 in Table 4 indicates the configuration used, while columns 2 to 5 show the mean HDinter values, as well as the mean, min, and max HDintra values for all the PUFs analyzed. The value of HDinter for each test system corresponds to the average Hamming distance between the responses of a given PUF and those of the PUFs implemented in other positions of the same test system. The mean, min, and max HDintra values are calculated as the average, minimum, and maximum, respectively, of the Hamming distances between the successive responses of the same PUF.

As it was logical to predict from the stability and entropy results obtained in the design characterization stage described in Section 4, the configurations that use Gray-code counters or/and lower bits present smaller values of HDintra, although sometimes at the expense of also reducing the values of HDinter, so it will be necessary to establish some kind of trade-off between reliability and robustness when selecting the PUF configuration for a given application. Table 4 also reveals the similar behavior with respect to the relative position of the compared ROs that we discussed earlier. On the other hand, the average values of HDintra obtained for any of the configurations are relatively high, which implies the use of complex ECCs that condition the size of the bit streams that must be used to generate keys of a required length. These circumstances justify the introduction of the challenge selection strategy described in the following section.

### 5.1. Challenge Selection Strategy

The strategy proposed in this work to increase the quality of the PUF consists in removing from its output the bits corresponding to the comparisons of RO-pairs that present a worse behavior from the stability point of view. To do this, those comparisons (challenges) whose results, after *t* consecutive applications of the challenge sequence, vary on a greater number of occasions with respect to the mode will be identified in a challenge selection mask to be excluded from the challenge sequence in the next calls to the PUF.

The algorithm included in the routine that performs the enrollment process for a specific instance of the PUF basically carries out the following tasks:

1. Stores the responses of the PUF for a number of calls defined by a user-defined parameter.
2. Calculates the mode of the values corresponding to each cycle of comparison (since in each cycle two pairs of ROs are compared and four bits are obtained, these values will be included in the [0,15] interval).
3. Evaluates, for each possible challenge, the number of times a response different from the one corresponding to the previously calculated mode is obtained, and ranks the possible challenges based on this data.
4. Generates the selection mask that identifies the *e* challenges (where *e* is also defined by a parameter of the enrollment routine) that will be eliminated, as well as the reference output of the PUF once these challenges have been discarded, which will be used to evaluate the reliability of the PUF.

The technique developed for the generation and use of the challenge selection mask was initially verified by software (with the help of the SDK functions) and later incorporated into the hardware implementation with the idea of optimizing the response time of the module.

**Figure 11:**
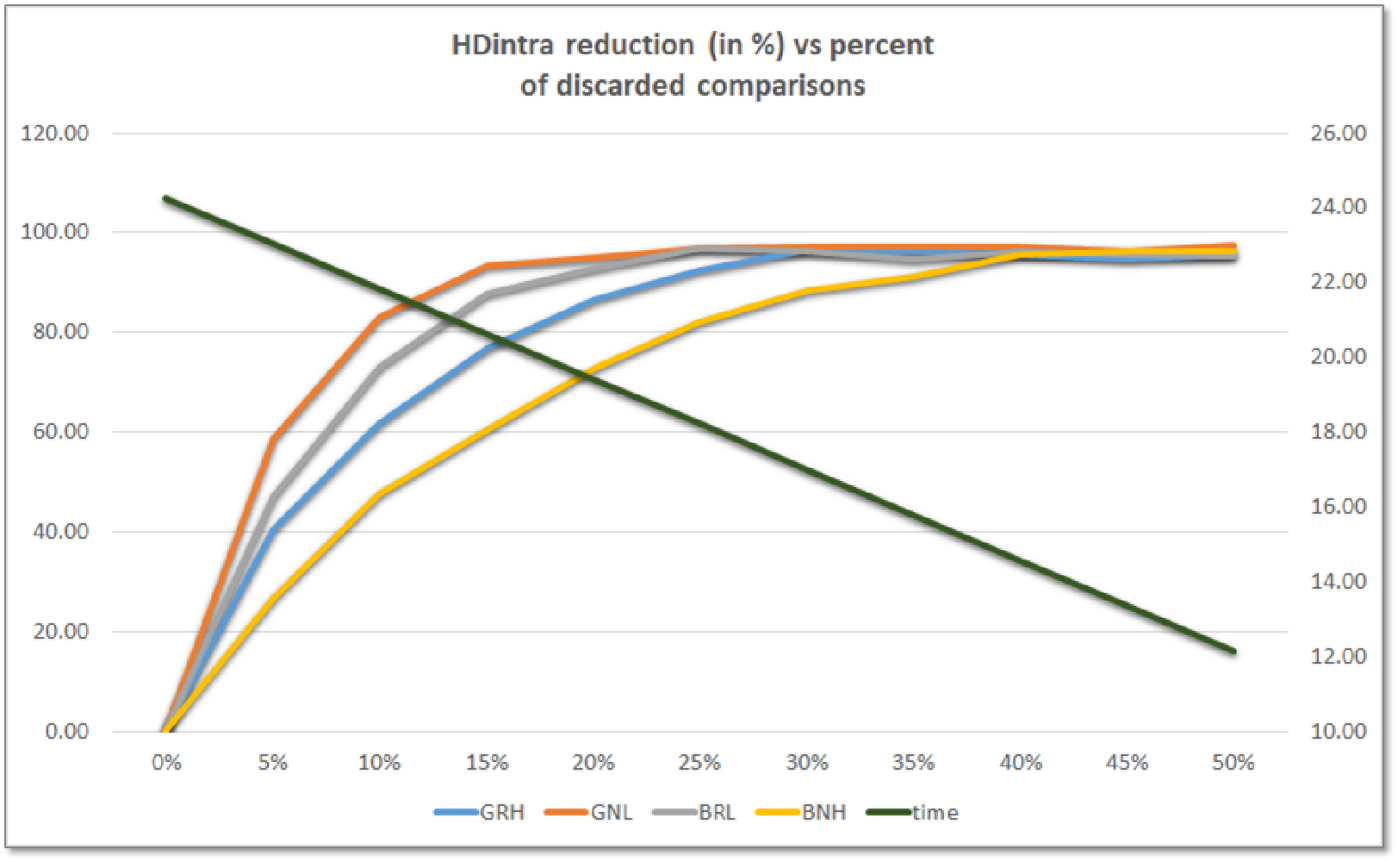
Reduction of HDintra and operation time as a function of the percentage of challenges discarded.

Figure 11 illustrates the percentage reduction of HDintra as a function of the percentage of challenges discarded for four of the possible configurations (the behavior is similar for the remaining four). The data represented in the graph correspond to the average values of the first five PUFs of the test system described above. As can be seen, they follow an exponential trend that causes HDintra to be reduced by more than 60% (BNH), 76% (GRH), 87% (BRL), and 93% (GNL) when the 15% of challenges are discarded.

Furthermore, the green line corresponding to the axis on the right of the graph, which shows the average time invested in invoking the PUF, illustrates how the hardware implementation of the challenge selection mechanism reduces the operation time by a percentage similar to that of challenges discarded, causing this to change from 24 ms when all challenges are applied to 18 and 12 ms when 25% and 50% of challenges are reduced, respectively.

**Figure 12:**
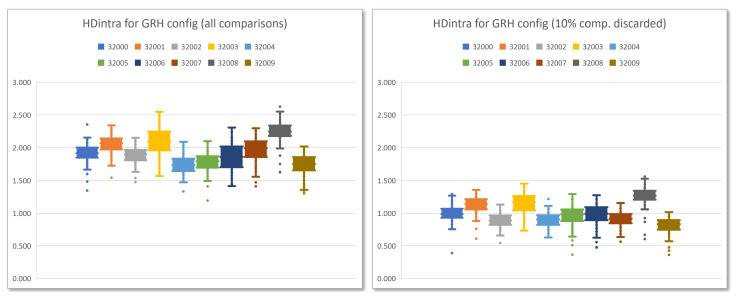
Distribution of HDintra values before (**left**) and after (**right**) discarding 10% of challenges for the ten PUFs included in the Pynq-Z2 test system.

The same behavior is also reflected in the box-and-whisker diagrams that appear in Figure 12, which show the distribution of the HDintra values corresponding to the ten PUFs of the test system when 100 series of 1000 calls (100,000 invocations) using the GRH configuration are made for each of the instances considering the 480 possible comparisons (left) and after performing an enrollment process on 1000 calls in which 10% of the comparisons are discarded (right).

**Figure 13:**
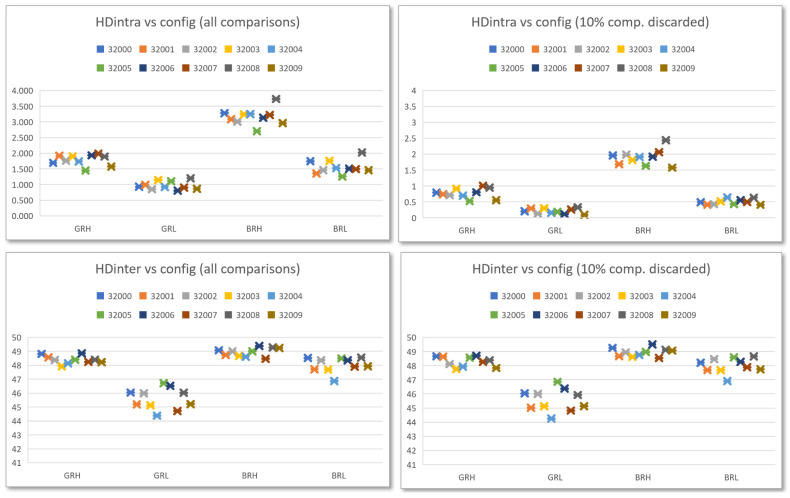
HDintra (**top**) and HDinter (**bottom**) values before (**left**) and after (**right**) discarding 10% of challenges for different configurations.

The reduction in the metric that determines the reliability when discarding the challenges that give rise to the most unfavorable comparisons is maintained in all possible configurations of the PUF. This is evident in the two diagrams on the top of Figure 13, in which the distribution of HDintra values is observed, before and after eliminating 10% of the challenges, for the four configurations that arise from considering the counter type and bit selection options. Data were obtained from the ten PUFs included in the Pynq Z2 test system using 1000 calls in the enrollment process and 10 runs of 1000 calls each (10,000 invocations) to calculate the Hamming distances.

A significant feature that is evident in the two bottom diagrams in Figure 13 is that the challenge selection strategy does not negatively affect the metric that determines the uniqueness of the PUF. As can be seen, HDinter values of the different PUFs are grouped into certain ranges, which depend on the configuration defined when invoking them, but are not affected when discarding one-tenth of the challenges.

The results obtained provide a series of clues when considering the practical application of the proposed PUF/TRNG module to generate IDs linked to the hardware of the device that incorporates it:

- The configuration parameters defined at run-time can be chosen to prioritize the reliability or robustness aspects of the PUF or to reach a trade-off between both.
- Increasing the number of challenges discarded in the enrollment process reduces the length of the bit stream provided by the PUF, but the decrease in the failure rate, evidenced by the smaller values of HDintra, allows the use of ECCs with a lower complexity for generating IDs of a certain length, so it is worth adjusting the selection strategy accordingly.
- Finally, the outputs provided by the different configurations of the PUF are strongly uncorrelated, making it possible to combine more than one of them to obtain longer bit streams.

### 5.2. Reliability and Uniqueness Evaluation

**Figure 14:**
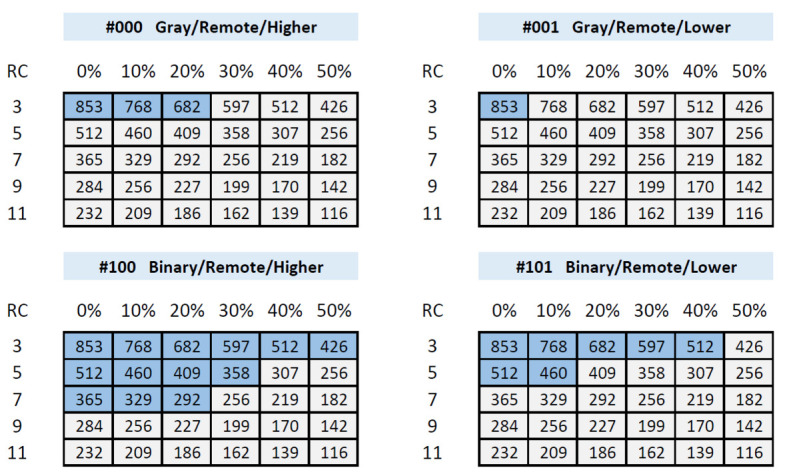
Pass/Fail in key retrieval for different configurations as a function of the percentage of discarded challenges and the ECC repetition factor (RC).

HDintra and HDinter metrics allow for estimating the repeatability and variability of the PUF responses from a statistical point of view. However, to validate the real usefulness of the developed module in combination with a Helper Data Algorithm (HDA) for the generation and recovery of secret keys, an extensive set of tests was carried out using ***puf_reliability*** and ***puf_uniqueness*** with the idea of determining the reliability and uniqueness of the IP module when used with PUF functionality. The results obtained for a PUF with a bank of 640 ROs and 14-bit counters implemented on the Genesys 2 board are represented in Figure 14, which illustrates by means of Pass/Fail tables the ability to obfuscate and recover the keys for four of the possible PUF configurations as a function of the percentage of discarded challenges and the repetition factor of the ECC used in the HDA.

The number that appears inside each cell indicates the length in bits of the generated key. In all cases, an enrollment process was carried out with 500 invocations to the PUF and an attempt was made to recover the secret 1000 times. Cells with a light background correspond to the cases in which the key was always recovered, while those with a dark background mark the situations in which there was at least one case in which the recovery was erroneous.

As shown in the tables, the use of Gray-code counters provides several alternatives to generate 512-bit keys using a single PUF configuration, but this is not possible when the output is taken from binary counters. The ability to generate and retrieve longer keys with the implemented PUF is also increased when the Lower option is chosen instead of the Higher one to select the bits that contribute to the output in each comparison. However, it is important to remember that, in this case, typical HDinter values decrease by 3 or 4 points, which could compromise the robustness of the PUF against certain types of attacks. A conservative trade-off to preserve the reliability and robustness of the PUF for generating a 512-bit key could consist, for example, of invoking the PUF twice with GRH and BRL configurations to obtain 256 bits in each.

Finally, to evaluate the uniqueness provided by the PUF, a series of tests were carried out to determine the number of times it was possible to recover the key in a PUF different from the one in which it was generated. The results obtained showed that keys could never be recovered, even using repetition factors much higher than those used in the reliability study in the obfuscation and recovery phases.

### 5.3. Changes in Operation Conditions

The results presented in the previous sections were obtained at room temperature and using the power supplies provided by the development boards that implemented the test systems. However, it is well known that changes in operating conditions (especially voltage and temperature) can negatively affect the performance of a PUF, sometimes making its use as a system security primitive inadvisable. For this reason, and in order to guarantee its quality before including it as an integral element of a hardware RoT, the proposed module was subjected to a series of additional tests to analyze, through the metrics described in the previous sections, how its performance is affected due to possible changes in the supply voltage and the operating temperature of the devices.

**Figure 15:**
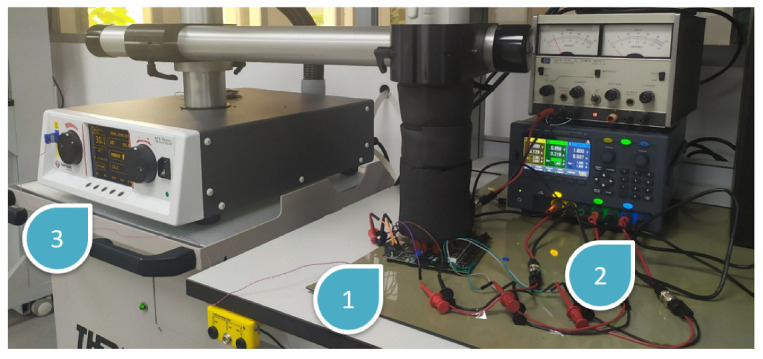
PUF characterization setup for voltage and temperature variations. 1. Development Board. 2. Power supply. 3. Temperature control system.

The experimental setup used to carry out these tests is shown in Figure 15. In addition to the Nexys A7 development boards used to implement the second of the test systems described in Section 2.4.1, it includes the multichannel power supply and the temperature control systems indicated in Table 5. Nexys A7 was selected because it is one of the few non-specialist FPGA development boards that offers the facility to apply external voltage supplies to its programmable logic, which is not possible in the case of the Pynq Z2 board.

**Figure 16:**
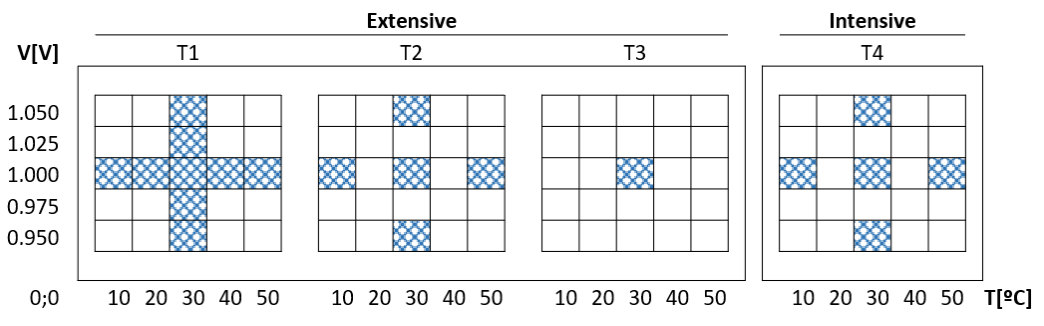
PUF voltage and temperature characterization space.

Considering the options that determine the codification of the counters (Binary or Gray) and the bits selected (Lower or Higher), four PUF configurations were subjected to variations in the operating conditions according to the Nexys A7 board manufacturer’s documentation [49]. Based on this information, a characterization space was established by adjusting the voltage and temperature values to the ranges represented in Figure 16. In addition to these two operating conditions, the number of boards and applications used for performance evaluation were also included as variables of the characterization space, which results in a space too large to be evaluated in its entirety. As an alternative, Figure 16 illustrates a compact characterization strategy that explores the characterization space under four different scenarios (T1–T4), where the colored cells represent the voltage-temperature combination evaluated in each case. This strategy includes two types of tests: extensive tests, to identify ranges and trends in PUF behavior over a short period of time; and intensive tests, to verify the stability of ranges and trends over time.

The characterization process was carried out using the application programs provided by the SDK with the aim of corroborating the behavior of the different PUF configurations included in this test system (***puf_enrollment***) and, especially, to evaluate the possible variations in the metrics that determine the reliability (***puf_HDintra*** and ***puf_reliability***) and uniqueness (***puf_HDinter*** and ***puf_uniqueness***) of the proposed module. The tests performed in each scenario (T1–T4) and the results obtained are summarized below, indicating the number of boards and applications involved in each case.

**T1—(1 Board-1 App)**

**Figure 17:**
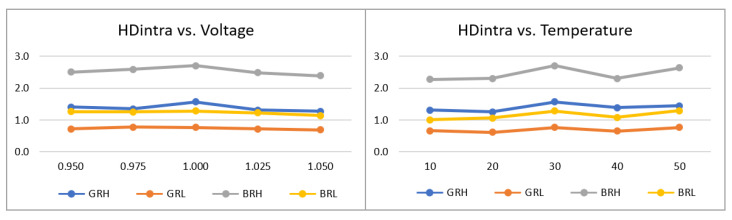
HDintra mean versus voltage (**left**) and temperature (**right**) variations for the test systems implemented in the Nexys A7 board.

The ***puf_enrollment*** application allowed verifying that the PUF behavior, for the four configurations considered (BRH, BRL, GRH, and GRL), is consistent when comparing the results of the HDintra metric evaluated in the enrollment process, presented in Figure 17, with those obtained for the test systems analyzed in previous sections. Additionally, it is observed that such values are stable against temperature and voltage variations in the characterization space, where the highest delta between the maximum and minimum values of HDintra for all configurations corresponds to 0.31 in temperature and 0.42 in voltage.

**T2—(1 Board-5 App)**

**Figure 18:**
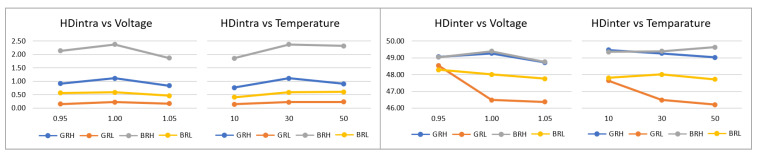
HDintra mean (**left**) and HDinter mean (**right**) versus voltage and temperature variations applying the challenge selection strategy on the test system implemented in the Nexys A7 board.

The effectiveness of the challenge selection strategy was also corroborated under different operating conditions by means of the ***puf_HDintra*** and ***puf_HDinter*** applications. In this case, the effect of eliminating 10% of the comparisons was evaluated, obtaining an average improvement in HDintra of 36.01% (BRH), 49.86% (GRH), 66.33% (BRL), and 81.09% (GRL) in voltage, and 35.81% (BRH), 50.62% (GRH), 67.34% (BRL), and 79.33% (GRL) in temperature, as shown in Figure 18. HDinter results are also consistent with previous results, and its response to variations in operating conditions resulted in a maximum average delta of 0.63 in voltage and 0.29 in temperature, both in the BRH configuration. There is an exception to this trend in the GRL configuration, whose HDinter response increases by 2.18 and 1.43 units as both voltage and temperature decrease, respectively, which represents an improvement from a uniqueness perspective.

Analyzing these metrics using the ***puf_reliability*** and ***puf_uniqueness*** applications with an ECC with a repetition factor equal to 9, it is observed that GRH, BRL, and GRL configurations present satisfactory results for the use of the PUF as ID generator.

**T3—(3 Boards-5 App)**

**Figure 19:**
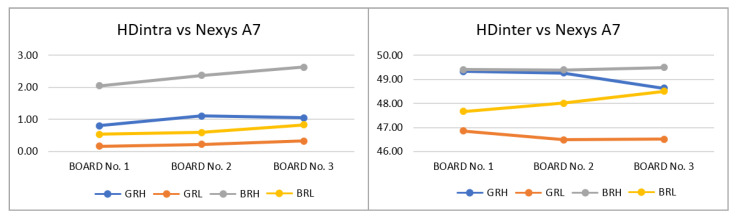
HDintra mean (**left**) and HDinter mean (**right**) under the same operation conditions, applying the challenge selection strategy on the test system implemented in three different Nexys A7 boards.

After analyzing the trends of PUF behavior against voltage and temperature fluctuations in a single board, the reliability and uniqueness features are verified in different boards by running the corresponding applications in the same test system under the same operating conditions. The results obtained show significant consistency between different boards, as can be seen in Figure 19, where a maximum delta in HDintra of 0.58 is identified for the BRH configuration and a maximum delta in HDinter of 0.84 in the BRL configuration. Since the tests were performed under a controlled environment, the estimated trends and ranges for voltage and temperature variations can be expected on different boards where the same PUF is implemented.

**T4—(1 Board-1 App)**

**Figure 20:**
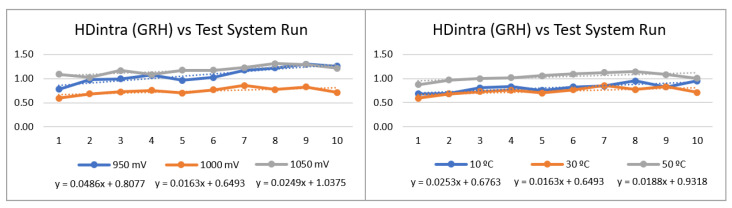
HDintra mean under voltage (**left**) and temperature (**right**) variations, applying the challenge selection strategy over two PUF instances of the test system implemented in the Nexys A7 board.

Finally, based on the trends and ranges identified for the four considered PUF configurations, two instances of the PUF with GRH configuration were selected to perform an intensive-type characterization. By running the test set ten times using the ***puf_HDintra*** application, the results show a slightly increasing trend at both voltage and temperature, as shown in Figure 20. Supported by a linear regression, it is confirmed that the slopes are positive, but it should be noted that all of them are in the order of hundredths. This allows inferring that, in the long term, the reliability of the PUF may be slightly negatively affected, making it advisable to repeat the enrollment process every certain period of time.

From these characterization results, we can conclude that the behavior of the PUF does present fluctuations in the metrics related to uniqueness and reliability in the temperature and voltage space considered, but the fluctuation ranges are not substantial with respect to the characterized results obtained for the reference operating conditions (30 °C and 1 V) in any configuration. Therefore, the quality of the analyzed PUF can be extrapolated within the ranges of the established characterization space to other test systems.

## 6. Performance Evaluation of the PUF/TRNG as an Entropy Source

The stability, probability and entropy metrics computed for the bits collected from the counters taking part in the RO comparisons, as discussed in Section 4, are also helpful in describing the operation of the proposed IP module as an entropy source. The metrics for the sign bit and the 14 bits of the counters in the two comparison blocks presented in Figure 9, when are analyzed from the perspective of TRNG functionality, demonstrate that the entropy increases in the same direction as the stability decreases and the probability approaches its ideal value. As a result, bit characterization highlights the need to choose the least significant bits when creating true random numbers.

The test system created on the Pynq Z2 board was used to gather the necessary bit streams to be subjected to a series of statistical tests in order to determine the level of randomness of RO-PUF/TRNG outputs. Using the ARM cores available on the Xilinx Zynq-7000 SoC, these tests can also be conducted on-line. The statistical evaluation procedure is carried out for each bit stream generated according to the NIST 800-22 [45] standard and the NIST 800-90b recommendation [46]. The latter describes the specifications for the entropy sources used by random bit generators, and the former establishes a set of 15 tests that determine whether binary data are uniformly random, ensuring that each bit has the same probability of taking either of the two possible states (0 or 1) and that it is statistically independent of the others.

### 6.1. True Random Number Generation Assessment

Taking into account the relative distance between the RO pairs (Remote or Nearby), the type of counter code (Binary or Gray), and the data extraction from the two counters independently in groups of one, two, and four LSBs, it was possible to derive 24 combinations that were characterized in order to identify the most adequate implementation to generate random numbers. The assessment strategy included the following stages:

1. A subset of statistical tests was used to analyze bit streams with lengths ≤500 bits in order to quickly identify a preliminary randomness characterization that distinguished the outcomes based on how many LSBs were used to construct them (one, two, and four). At this point, the results allowed us to draw the conclusion that only bit streams built with one and two LSBs had adequate levels of randomness.
2. The randomness of new bit streams constructed with the concatenation of the two LSBs from each counter was assessed using the same subset of tests, and the results obtained allowed us to conclude that the concatenation approach is better suited to the objective of collecting the maximum number of bits per RO-pair competition with a good level of randomness. Consequently, the combinations derived considering the concatenation approach are reduced from 24 to four, since only the parameters regarding the relative distance between pairs of ROs and the type of counter code are now involved.
3. The statistical characterization of the four possible combinations was carried out by applying the complete set of tests of the standard to bit streams with a length equal to 10^6^ bits, concluding that the bit streams based on the Binary/Remote IP configuration consistently pass all the statistical tests and exhibit uniform performance along the programmable logic, demonstrating a high level of randomness. Although the remaining three combinations (Binary/Nearby, Gray/Nearby, and Gray/Remote) also have fairly homogeneous performance throughout the FPGA, they do not pass all 15 tests of the standard.
4. The bit streams of the latter combinations fail a specific subset of tests whose general approach is based on analyzing the ratio of zeros and ones in a sequence of random bits, which ideally should be 50% for any given case. Therefore, in order to increase the degree of randomness of these configurations and the ability of the IP to generate true-random numbers, the gathered bit streams were put through post-processing to lessen their bias. For this purpose, the von Neumann and XOR correctors were implemented in software, and the post-processed data were reassessed with the full set of tests defined by the NIST standard. The results allowed us to conclude that the von Neumann corrector improves the rate of tests passed by the bit streams but not in its totality, while the XOR corrector allows the three configurations to reach 100% of tests passed.

Table 6 details the evaluation findings for the four TRNG configurations previously found to be capable of passing the NIST 800-22 standard statistical tests. The minimum pass rate required by the standard for each test using a sample size of 100 binary sequences is 96. The Random Excursion tests are typically evaluated under a separate threshold, but in this study, the data were normalized to the same scale to make them more comprehensible. Columns 2 through 5 include the pass rate for each test, which was calculated as the average of 10 IP module implementations. The minimum pass rate is 96, the median is 99, and the overall average pass rate for these findings is 98.27. The test pass rate attained in other comparable works is shown in columns 6 through 13.

Comparatively, the minimal pass rates among our four TRNG configurations are greater than or equivalent to 40% of the statistical findings shown in [50,51,52], and greater than or equivalent to 33% of all cited works. These findings demonstrate that the four TRNGs derived from the original PUF design possess a sufficient level of randomness and are state-of-the-art.

**Table 6:** Average test pass rate of the four TRNGs against those proposed in other related works using the NIST 800-22 statistical test suite.

Test	This Work				[53]		[51]		[40]	[52]	[50]	[54]
	XOR			RAW	TERO	COSO	A	B				
	GC	GF	BC	BF								
Frequency	99	98	99	97	99	99	97	100	95	98	96	100
Block Frequency	99	98	98	99	99	99	100	99	95	99	97	98
Cumulative Sums ${}^{★}$	98	98	98	97	99	99	98	100	95	98	97	99
Runs	99	99	99	99	99	99	96	96	100	98	99	100
Longest Run	99	99	99	98	98	99	100	99	100	98	98	99
Rank	98	98	98	98	99	99	100	100	100	98	98	100
FFT	98	98	99	98	98	99	97	98	100	98	98	100
Non-overlapping Template ${}^{★}$	98	98	99	98	98	99	99	98	100	98	100	100
Overlapping Template	98	98	98	98	99	99	100	100	100	98	99	98
Universal	98	98	98	98	99	99	97	99	95	99	99	99
Approximate Entropy	98	98	98	97	99	99	100	100	100	99	100	99
Random Excursions ${}^{★}$	99	99	99	98	99	99	100	98	100	99	100	98
Random Excursions Variant ${}^{★}$	99	98	99	97	99	99	99	100	100	99	100	99
Serial ${}^{★}$	98	98	98	98	99	99	98	100	100	98	100	99
Linear Complexity	99	98	98	98	99	99	96	96	100	98	99	98
μ	98.5	98.2	98.5	97.9	98.8	99.0	98.5	98.9	98.7	98.3	98.7	99.1

${}^{★}$ Tests that include multiple sub-tests. TERO: Transition Effect RO. COSO: Coherent Sampling RO. A: Latched RO without feedback. B: Latched RO with feedback.

### 6.2. PUF Validation as Entropy Source

The entropy source used to derive the four TRNG configurations has been validated following the process established in NIST 800-90b recommendation, according to which any bit stream under assessment requires $10^{6}$ bits and every collected bit stream should be built by concatenating groups of at least $10^{3}$ bits. Since the maximum number of bits that can be generated in a single call by the IP modules included in the test systems is 1920 due to the selected RO bank size and the amount of bits selected from each comparison (480 × 4), the data for the validation process were collected in groups of $10^{3}$ bits and concatenated after $10^{3}$ IP calls.

This process includes the execution of statistical tests through two stages known as Entropy Estimate and Restart. The first stage allows, on the one hand, to estimate an entropy value for the source by evaluating outputs that have been collected during the continuous operation of the system and, on the other hand, to distinguish whether the output samples are Independent and Identically Distributed (IID) or not (Non-IID). The second stage re-evaluates the entropy estimate in the corresponding track (IID or Non-IID) using a single bit stream composed of outputs from $10^{3}$ restarts of the system (as mentioned before) to ensure its quality.

Using 100 bit streams to perform the validation process, the statistical results of the Entropy Estimate stage reflect a fairly high pass rate for each of the tests that constitute the IID-track assumption for the samples (Chi-square Independence Test-99%, Chi-square Goodness-of-fit Test-99%, LRS Test-100%, and IID Permutation-99%). Although these values are significantly high, they do not fully satisfy the assumed track; therefore, it was decided to continue the validation process assuming the Non-IID-track.

Table 7 shows the average entropy estimated by each of the statistical tests that make up the Non-IID-track assumption. According to the documentation, the entropy value estimated for the source corresponds to the lowest result within the tests; therefore, in the four configurations presented in the table, the entropy values correspond to the result of the Compression Estimate test.

The Restart stage performs the same statistical tests used in the Non-IID track for the Entropy Estimate stage and adds a Sanity Check test that verifies the ratio of 0s and 1s. The results show that the collected data present a ratio close to 50% in all cases; thus, the Restart stage is passed in its totality, and the entropy value is updated based on the statistical results of the bit stream constructed for this stage.

The successful execution of the two stages (Entropy Estimate and Restart) validates the entropy source according to the NIST recommendation and shows that, as long as the predicted entropy is roughly constant, no created bit stream will remain stagnant at a single value, the zero-to-one ratio will be about 50%, and the ability to predict future sequences after starting the system does not depend on knowledge of previous sequences.

In order to have a method to detect significant changes in behavior as a source of entropy, the Adaptive Proportion and Repetition Count health checks tests, proposed within the NIST recommendation, were also included in the software to monitor the quality of the design.

To summarize, Table 8 shows that the proposed module has features that allow the implementation of four different TRNGs based on the location of competing rings and counter code type. All four TRNGs have passed the NIST-800-22 standard tests, proving that they can generate truly random numbers. It is worth noting that the Binary/Remote configuration does not need any post-processing stage to generate random bit streams, while the other three configurations need to undergo XOR bit correction to pass all the standard tests. This post-processing stage reduces the number of effective bits by 50%.

## 7. Conclusions

The use of a root of trust linked to the hardware on which it is implemented constitutes an efficient alternative to increase the security of IoT devices, avoiding device counterfeit and software attacks with the inclusion of implementations of cryptographic algorithms at the hardware level. This work describes the design of a basic primitive for a hardware root of trust, which offers dual functionality as a physical unclonable function that provides identifiers linked to the devices and as a source of entropy capable of generating true random numbers.

The PUF/TRNG module takes full advantage of the structure and features of the Xilinx Series-7 and Zynq-7000 programmable devices to provide a compact implementation, suitable to be incorporated into resource-constrained IoT devices. Performing two simultaneous comparisons between two pairs of elements of the RO bank, as well as the possibility of using configuration options to select the type of counter, the relative position of the ROs compared, and the bits selected in each comparison cycle, allow for a bit rate per area higher than other proposals in the literature. In addition, the inclusion of a challenge selection mechanism, to discard after an enrollment phase the comparisons that most negatively affect the repeatability of the PUF response, allows a drastic reduction of the Hamming distance between outputs of successive PUF invocations, increasing in the same proportion the reliability of the system.

To offer hardware-based IoT security solutions, the RO-PUF/TRNG design was encapsulated as a parameterized IP module, for which the designer can define the size and position of the RO bank, the length of the counters, and the operation mode according to the characteristics of a particular application. The IP was also provided with a standard communication interface based on the AXI4-Lite bus to facilitate its integration with general-purpose processors usually available in embedded systems.

The work also provides a software development kit that includes a rich set of low- and high-level drivers and C-coded functions intended to facilitate module operation as an ID generator or entropy source, as well as to evaluate its performance for on-line characterization and monitoring purposes. Using this software, in combination with test systems that include different instances of the IP module and use ARM and MicroBlaze processors, an exhaustive set of tests has been carried out to evaluate the metrics that determine its quality when acting as PUF and TRNG.

The results obtained show that the module behaves as a TRNG that complies with the standard and recommendations proposed by NIST for different run-time options defined by the user. These options also allow selecting a suitable trade-off between robustness and reliability when the IP is used as a PUF, and can even be combined to increase the size of the output bit stream in applications that require obfuscating and recovering a secret or cryptographic key. The dependence of the main quality indices of the module against changes in operating conditions is also analyzed in the work, in which routines are also provided to carry out an on-line monitoring strategy to detect possible risk situations in terms of system security.

Finally, the software and test systems developed in this work provide a useful tool for the evaluation and optimization of PUF/TRNG designs in future works. They can also be adapted to evaluate other designs with different structures and technologies, which will be useful to researchers and practitioners working in the field of hardware security.

## Figures and Tables

**Table 1:** Resource utilization of PUF/TRNG cores in Spartan-7, Artix-7, Zynq-7000, and Kintex-7 devices with 14-bit counters and different number of ROs.

Device	ROs	LUTs	Registers	Slices	Block RAMs
XC7S50-CSGA324	360	1458 (4.47%)	248 (0.40%)	478 (5.87%)	1.0 (1.33%)
XC7A100T-CSG324	480	1869 (2.95%)	229 (0.18%)	549 (3.46%)	0.5 (0.37%)
XC7Z020-CLG400	480	1870 (3.52%)	229 (0.22%)	545 (4.10%)	0.5 (0.36%)
XC7K325T-FFG900	640	2300 (1.13%)	233 (0.06%)	640 (1.26%)	0.5 (0.11%)

**Table 2:** Resource utilization for different test systems implementing 32-bit AXI4-Lite interfaces.

Test System	Mode	LUTs	Registers	Slices	Block RAM
Pynq-Z2	C *	19,673 (36.98%)	4639 (4.36%)	5799 (43.60%)	5 (3.57%)
	O ^†^	19,591 (36.83%)	4659 (4.38%)	5794 (43.56%)	5 (3.57%)
Nexys A7	C	20,917 (32.99%)	12,202 (9.62%)	6946 (43.82%)	71 (52.59%)
	O	20,882 (32.94%)	1212 (9.63%)	6896 (43.51%)	71 (52.59%)

* Characterization mode. ^†^ Operation mode.

**Table 3:** Main mid- and high-level functions included in the SDK.

Function	Task
PUF_createMMIOWindow	Create memory-mapped IO window for PUF/TRNG registers
PUF_applyChallenges	Reset, configure and start PUF/TRNG operation
PUF_readOutput	Read PUF/TRNG results from the output memory
PUF_enrollment	Generate PUF output reference and challenge selection mask
PUF_writeChallegesMask	Write the challenge selection mask
PUF_HD	Calculate mean, minimum, and maximum Hamming distance

**Table 4:** HDinter mean and HDintra mean, min, and max values for different configurations of the PUFs included in the test systems implemented on the Pynq Z2 board.

Configuration	HDinter_mean	HDintra_mean	HDintra_min	HDintra_max
Binary/Remote/Higher	48.95	3.16	1.51	5.42
Gray/Remote/Higher	48.39	1.79	0.52	2.81
Binary/Remote/Lower	48.04	1.56	0.47	3.18
Gray/Remote/Lower	45.59	0.97	0.26	1.88
Binary/Nearby/Higher	48.94	3.20	1.77	5.52
Gray/Nearby/Higher	48.40	1.83	0.73	3.33
Binary/Nearby/Lower	47.99	1.60	0.57	3.07
Gray/Nearby/Lower	45.54	1.00	0.31	1.82

**Table 5:** Features of the test system for the temperature and voltage characterization process.

No.	Instrument	Reference	Qty
1	Development Board	Nexys A7	3
2	Power supply	Keysight e36312A	1
3	Temperature control system	Thermonics ATS-505-S-2	1

**Table 7:** Entropy estimation of four TRNGs using the statistical tests included in NIST 800-90b recommendation for Non-IID-track.

Test	XOR			Raw
	GC	GF	BC	BF
Most Common Value Estimate	0.995915	0.995351	0.995543	0.993609
Collision Estimate	0.917535	0.905876	0.896818	0.895582
Markov Estimate	0.999247	0.999097	0.997907	0.998003
Compression Estimate	0.836274	0.830815	0.882088	0.843385
t-Tuple Estimate	0.931433	0.921623	0.921623	0.939780
LRS Estimate	0.919974	0.996316	0.989705	0.986412
MultiMCW Prediction Estimate	0.998528	0.998482	0.996301	0.994446
Lag Prediction Estimate	0.995447	0.996420	0.995430	0.994662
MultiMMC Prediction Estimate	0.995224	0.996530	0.994583	0.996677
LZ78Y Prediction Estimate	0.997862	0.998061	0.996336	0.994705

**Table 8:** Summary of TRNG randomness assessment results and their validation as a source of entropy.

PUF Configuration	Post-Process	NIST 800-22	NIST 800-90b
Binary/Remote	–	Pass(15/15)	Validated
Binary/Nearby	XOR	Pass(15/15)	Validated
Gray/Remote	XOR	Pass(15/15)	Validated
Gray/Nearby	XOR	Pass(15/15)	Validated

## Data Availability

Not applicable.

## Abbreviations

The following abbreviations are used in this manuscript:

ASIC	Application-Specific Integrated Circuit
AXI	Advanced Extensible Interface
BRAM	Block Random-Access Memory
CLB	Configurable Logic Block
DRAM	Dynamic Random-Access Memory
ECC	Error-Correcting Code
FPGA	Field-Programmable Gate Array
FSM	Finite State Machine
GUI	Graphical User Interface
HDA	Helper Data Algorithm
HW	Hardware
IC	Integrated Circuit
ID	Identifier
IID	Independent and Identically Distributed
IoT	Internet of Things
IP	Intellectual Property
LUT	Look-Up Table
LSB	Least Significant Bit
MSB	Most Significant Bit
NIST	National Institute of Standards and Technology
PL	Programmable Logic
PS	Processor System
PUF	Physical Unclonable Function
PYNQ	Python Productivity for Zynq
RO	Ring Oscillator
RoT	Root of Trust
SoC	System on Chip
SDK	Software Development Kit
SRAM	Static Random-Access Memory
SW	Software
TRNG	True-Random Number Generators
